# Comparing Antecedents of Chinese Consumers’ Trust and Distrust

**DOI:** 10.3389/fpsyg.2021.648883

**Published:** 2021-05-13

**Authors:** Dan Zhao, Xiaofeng Shi, Sheng Wei, Junsheng Ren

**Affiliations:** ^1^Business School, Jilin University, Changchun, China; ^2^Lecturer of Home Economics, College of Humanities, Jilin Agricultural University, Changchun, China; ^3^School of Business, Nanjing Audit University, Nanjing, China; ^4^School of Management, Harbin University of Commerce, Harbin, China

**Keywords:** trust, distrust, critical incident technique, Chinese consumers, retailing

## Abstract

To help retailers gain consumers’ trust, many studies have investigated antecedents of consumer trust. However, distrust, a concept closely related to trust, has attracted only sporadic research attention. As a result, whether factors that increase consumer trust can eliminate consumer distrust is unclear. To deepen understanding of trust and distrust, this study applies the critical incident technique to identify and compare the antecedents of trust and distrust of Chinese consumers. The results show that the antecedents of distrust differ from those of trust, indicating different formulation mechanisms of both. Therefore, on the one hand, retailers should pay attention to increasing consumer trust, and on the other hand, they should develop marketing activities to reduce consumer distrust.

## Introduction

Trust has attracted a great deal of research attention in many fields in the past decades, including marketing, business ethics, management, politics, and psychology (e.g., Morgan and Hunt, 1994; Sirdeshmukh et al., 2002; Pan and Zinkhan, 2006; Keller et al., 2015; Roy et al., 2015; De Jong et al., 2016; Pappas, 2016; Pirson et al., 2017; Martin and Torcal, 2019; Su et al., 2019). To help retailers gain consumers’ trust, many studies have investigated antecedents of consumer trust and empirically examined a plethora of factors, such as perceived familiarity, perceived similarity, perceived control, consumers’ personality, and atmosphere (e.g., Walczuch and Lundgren, 2004; Johnson and Grayson, 2005; Chen and Dibb, 2010). However, distrust, a concept closely related to trust, has attracted only sporadic research attention (e.g., Leslie, 2003; Steindl and Jonas, 2015; Ryan, 2017; Aghakhani and Main, 2019; Alasfour, 2019). The disproportionate academic attention to the two concepts is largely due to the assumption that trust and distrust are two sides of the same coin, or two bipolar concepts along a continuum (e.g., Rotter, 1971; Gurtman, 1992) and, as such, that retailers need to focus only on trust, as marketing activities devoted to increasing trust can automatically decrease distrust. By contrast, other studies have shown that trust and distrust are distinct constructs, such that the absence of trust does not necessarily mean the presence of distrust, or vice versa (Lewicki et al., 1998; Cho, 2006; Chang and Fang, 2013; Van De Walle and Six, 2014; Kujala et al., 2016; Gefen et al., 2020). To add to this conversation, this study attempts to compare the antecedents of trust and distrust of Chinese consumers. In doing so, it makes three contributions to practice and theory.

First, the findings can help managers develop marketing tactics to build consumers’ trust and prevent distrust. Specifically, if the antecedents of trust and distrust overlap to a great extent, managers only need to try to increase consumers’ trust. Otherwise, they need to manage the coexistence of trust and distrust by working to build and maintain consumers’ trust while decreasing consumers’ distrust. Unsuccessful management of the coexistence of trust and distrust may cause consumers who did not initially distrust firms *per se* not to trust them at all and, thus, to avoid their marketing activities (Chaudhuri and Holbrook, 2001; Harris and Goode, 2010; Bianchi et al., 2017; Giboa et al., 2019). Knowing the antecedents of trust and distrust can help retailers directly formulate and implement marketing activities to build and maintain good relationships with consumers, which is the primary goal of this research.

Second, our findings help enrich the understanding of the relationship between trust and distrust. As Lewicki et al. (1998, p. 440) posit, if trust and distrust are two distinct constructs, different elements should “contribute to the growth and decline of trust and distrust.” Following this logic, many studies have examined and compared the asymmetrical influences of antecedents on trust and distrust (e.g., Cho, 2006; McKnight and Choudhury, 2006; Ou and Sia, 2010; Connelly et al., 2012). These studies usually incorporate a set of preselected antecedents into models and empirically examine whether the effect sizes of the influence of antecedents on trust and distrust are symmetrical. Admittedly, doing so provides insights into whether the antecedents influence trust and distrust to the same extent. However, one disadvantage of this quantitative method is the possibility of overlooking some antecedents in models, and this omission might bias the effect sizes of the influence. Therefore, more qualitative evidence is required to test whether “the elements” contributing to trust and distrust are different. To achieve this goal, we employ the critical incident technique (CIT) to exhaust and compare antecedents of consumers’ trust and distrust in physical retail stores. In addition, our study responds to recent calls to examine the relationship between trust and distrust in various marketing contexts, as the two constructs have been predominantly examined in the field of online commerce (McKnight and Choudhury, 2006; Chang and Fang, 2013). To answer these calls, we chose physical retail stores as our research setting to examine whether consumer trust and distrust have different antecedents.

Third, this study is based on Chinese consumers, and as such, the findings offer a unique perspective to understand trust and distrust. Because an institutional trust environment is not fully developed in China (Luo et al., 2008), Chinese consumers are likely to have different propensities to trust and distrust a retail store than those of consumers from developed countries with more mature institutional trust environments. Therefore, understanding Chinese consumers’ trust and distrust advances extant literature on trust and distrust.

In the remainder of the article, we first review the literature on trust and distrust and then detail the process of CIT by which we identify the antecedents of trust and distrust. We then explain and discuss the results. Finally, we present the implications and limitations and provide directions for future research.

## Literature Review

### Definition of Trust and Distrust

Trust has been defined from the perspectives of personality traits, beliefs, intentions, motivations, outcomes, and formation mechanisms, among others. In terms of beliefs, trust refers to the expectation held by a trustor that a trustee will behave dependably, ethically, and honestly (Gefen et al., 2003). In terms of motivation, trust involves “the willingness of a party to be vulnerable to the actions of another party based on the expectation that the other will perform a particular action important to the trustor, irrespective of the ability to monitor or control that other part” (Mayer et al., 1995, p. 712). In terms of personality traits, trust emphasizes the propensity to trust (Kong and Hung, 2006). Our study adopts Sirdeshmukh et al. (2002, p. 17) definition of consumer trust in a retailer as “expectations held by consumers that a retailer is dependable and can be relied on to deliver offerings on his/her promises.”

Because research attention to distrust is rather limited, many definitions of distrust are based on reciprocal words of definitions of trust. For example, Lewicki et al. (1998) define trust as the positive expectation a trustor holds of a trustee and define distrust as the negative expectation a trustor holds of a trustee. Specifically, when a trustor has high trust in a trustee, the trustor feels safe, secure, hopeful, confident, and comfortable (Lewicki and Brinsfield, 2009; Kujala et al., 2016). By contrast, distrust in the trustee makes the trustor feel insecure, worried, fearful, suspicious, and vigilant (Lewicki and Brinsfield, 2009; Kujala et al., 2016). As the presence of fear, insecurity, and worry does not necessarily mean the absence of safety, hope, and confidence, investigating the antecedents of trust and distrust separately is necessary (Schul et al., 2008).

This study focuses particularly on Chinese consumers’ trust and distrust because the Chinese market is characterized by an inferior environment for developing institutional trust (Steinhardt and Delhey, 2020). Chinese consumers’ institutional trust is rather low, due to, for example, a few notorious scandals on food safety and the embezzlement of the Red Cross Society of China, which have led to consumers’ distrust of administrative, legal, and/or societal institutions (Cheng, 2016). If they do not believe that legal institutions will provide adequate assistance when they are deceived by retailers, consumers may be vigilant and more alert when making purchase decisions. In this sense, gaining consumers’ trust and decreasing their distrust should help build relationships with consumers, but doing so is an extremely challenging task for retailers. Therefore, in this study we investigate what leads to Chinese consumers’ trust or distrust given unfavorable environments of institutional trust, to provide retailers with suggestions on how to build relationships with consumers.

### Antecedents of Trust and Distrust

Although researchers have defined trust in somewhat different ways, they have largely reached a consensus that the antecedents of trust primarily center on trustees’ ability/competence, benevolence, and integrity (Ali and Birley, 1998; Mayer et al., 1995). Ability/competence refers to skills and expertise that enable trustees to exert influence within a specific field (Mayer et al., 1995). Benevolence captures the extent to which a trustor believes that a trustee wants to do good regardless of the trustee’s own profits, and integrity refers to the extent to which a trustor believes that a trustee acts in accordance with acceptable social norms (Mayer et al., 1995; Palmer and Huo, 2013). As trust is a multidimensional concept, a trustor may trust a trustee’s ability but distrust his or her benevolence. For example, a person may trust his or her colleague’s ability in the workplace but think that he or she is selfish (e.g., distrust in benevolence), suggesting the coexistence of trust and distrust. Turning to the field of retailing, we propose a framework of antecedents of consumer trust based on previous studies, including four aspects: core product-related, service-related, third-party-related, and overall store-related elements.

Core product-related elements involve consumers’ overall assessment of the performance of core products and services provided by retailers (Johnson and Grayson, 2005). Because core products and services are the focal objects during the purchase process, perceived value of products and services should be critical in building consumer trust. If a consumer had a satisfactory experience with the performance of products purchased in a store, it is likely that he or she will trust the store (Tax et al., 1998).

Service-related elements include elements associated with consumers’ overall assessment of the process of interacting with salespeople and the supporting services that facilitate the delivery of core products and services (Auh, 2005). Although services are provided during the whole purchase process, service-related elements in this study include only characteristics of supporting service, such as shipping, free returns, after-sale services, protection of privacy, and security. Elements related to core services are categorized into core product-related elements. For example, offering delicious and healthful food is a service a restaurant provides, but this service is categorized into core product-related rather than service-related elements. The elements supporting services function as external clues to reflect a store’s competence and ability to serve consumers, so they should influence consumers’ trust (Kim et al., 2005; Guenzi et al., 2009). In addition, salespeople play an important role in delivering products and services because they have direct interactions with consumers (Tax et al., 1998; Kim et al., 2011). As a result, consumers’ trust in a physical store largely depends on their evaluations of interactions with salespeople (Sun and Lin, 2010). With a satisfactory interaction, consumers can gain confidence and social benefits, which in turn enhance their relationship with the store (Gwinner et al., 1998). Salespeople’s positive characteristics, such as expertise, likability, benevolence, and competence all influence consumers’ trust in a store (Doney and Cannon, 1997; Guenzi and Georges, 2010; Sun and Lin, 2010; Kim et al., 2011).

Third-party-related elements refer to recognition from an objective third party, such as consumer associations, TRUSTe, the media, and the like (Cook and Luo, 2003). When consumers purchase products, they are subject to potential risks such as defective products, overpricing, and so on (Wang and Hsiao, 2012), and recognition from a third-party institution can decrease their perceived risks (Benassi, 1999). For example, local governments in China regularly issue certificates to eligible companies to recognize their reliability, dependability, and trustworthiness. Therefore, we argue that seeing such certificates may reduce consumers’ perceived risks and uncertainty, because their trust in local governments can be transferred to the company through these certificates (Stewart, 2003; Cheung and Lee, 2006).

Last, overall store-related elements refer to consumers’ overall evaluation of a store rather than specific products, salespeople, or supporting services (Orth and Green, 2009). For example, consumers can formulate a holistic image of a store based on overall atmosphere, word of mouth, in-store posters, corporate social responsibility, and other aspects (e.g., Siau and Shen, 2003; Aiken and Boush, 2006; Lunardo and Mbengue, 2013). Consumers can develop trust in retailers through the management policies and practices that govern exchanges. To evaluate a store’s trustworthiness of management policies and practices, consumers may use both intrinsic cues (i.e., product- and services-related elements) and extrinsic cues, which are captured as overall store-related elements in this study. For example, when a store has a comfortable atmosphere, consumers might trust the store because they may associate it with operational excellence (Orth and Green, 2009).

As mentioned previously, in sharp contrast with trust, distrust has received only sporadic research attention in terms of its antecedents. A limited number of studies on antecedents of distrust have found that the same antecedents asymmetrically influence both trust and distrust (Wang and Benbasat, 2008; Xiao and Benbasat, 2010; Connelly et al., 2012). For example, consumers’ expertise and the usefulness of information increase trust but do not decrease distrust (Chang and Fang, 2013). In addition, Cho (2006) found that the hindering effect of competence on distrust is greater than its enhancing effect on trust, while Simon and Cagle (2015) found the opposite result. Moreover, Chang and Fang (2013) showed that a trustee’s expertise influences only trust, not distrust. In addition, studies based on functional magnetic resonance imaging (fMRI) indicate that trust and distrust should be caused by different antecedents because they are associated with the activation of different brain areas (Dimoka, 2010). Specifically, fMRI results show that trust correlates with the caudate nucleus, the anterior paracingulate cortex, and the orbitofrontal cortex while distrust is linked to the amygdala and the insular cortex (Dimoka, 2010; Fett et al., 2014). As such, trust and distrust should be caused by different characteristics of a retailer.

In summary, people are more sensitive to distrust than trust because the evolutionary process has made them more aware of negative than positive objects and situations (Baumeister et al., 2007; Lumineau, 2017). As a result, the impact of distrust on reducing consumers’ positive responses is more prominent than that of trust on enhancing their positive responses (Cho, 2006). In other words, when consumers trust a retailer, they may not necessarily purchase from it, but when they distrust a retailer, they are quite likely to boycott it. Therefore, comprehensively comparing the factors that lead to trust and distrust is imperative so that marketers can develop pertinent measures to increase consumer trust and decrease distrust. To achieve this goal, this study uses CIT to uncover and compare antecedents of consumers’ trust and distrust in retailers.

## Research Design and Results

### Data Collection and Sample

To uncover and compare antecedents of trust and distrust, we employ the CIT, a tool that has been used extensively in exploratory studies in the field of marketing (Stokes, 2002; Arnold et al., 2005; Harmeling et al., 2015). CIT is a qualitative data collection procedure through which researchers collect, classify, and analyze data to gain an understanding of the impacts of critical incidents on key variables of interest (Hopkinson and Hogarth-Scott, 2001; Gremler, 2004). Critical incidents in this study refer to out-of-the-ordinary events during an interaction that customers perceive or recall as unusually salient to cause their trust or distrust (van Doorn and Verhoef, 2008). In service research, critical incidents are usually collected by asking research participants to tell a story about an experience relevant to the phenomenon being investigated (Gremler, 2004).

In this study, we use CIT because it has four advantages over other research methods. First, CIT does not confine observations to a limited set of variables, as participants are given opportunities to write their own experiences in their own words (Gremler, 2004). As we discussed previously, one theoretical gap in extant studies is that they quantitatively examine a series of preselected antecedents of trust vs. distrust; doing so may bias the effects of these antecedents on trust or distrust. However, compared with other research methods, CIT allows researchers to identify antecedents of trust and distrust more extensively and to develop a comprehensive framework. Second, CIT allows participants to determine which incidents are the most critical for the variables of interest. Specifically, we attempt to uncover key antecedents of trust, which should be reflected in consumers’ most memorable shopping experiences that engendered trust. More importantly, CIT is especially appropriate to identify antecedents of consumers’ distrust, because even one negative event can lead to a considerable decrease in consumers’ evaluation rating, which thus leads to distrust (van Doorn and Verhoef, 2008). Third, CIT is particularly effective in developing conceptual structures that can be tested in subsequent research (Walker and Truly, 1992). Because the antecedents of Chinese consumers’ distrust in retail stores are under researched, the CIT is an appropriate method to explore distrust’s antecedents. In this sense, we argue that CIT is applicable in comprehensively identifying and comparing antecedents of trust and distrust. Fourth, CIT is “a powerful tool which will yield relevant data for practical purposes of actioning improvements and highlighting the management implications” (Chell and Pittaway, 1998, p. 24). As alluded to previously, the primary goal of this research is to help retailers formulate and implement marketing activities to build and maintain good customer relationships. CIT enables the collection of a rich source of relevant, unequivocal, and concrete data and information that can suggest improvement for retailers.

We invited 232 undergraduate students at a public university in China to recall and write about two stories in which they experienced trust and distrust in a physical retail store, respectively. According to Gwinner et al. (1998), students are appropriate research subjects in retail research. To ensure that all students completely understood the question, a sample answer was given before they began writing their stories. We asked the students to recall and describe the experience in the manner of who, what, when, where, why, and how. The whole process was under supervision and lasted 45–60 min on average. We judged 13 samples as unusable because of incomplete information or unrecognizable writing, which left 219 samples. Of the remaining participants, 41% were male and 59% were female. From the stories, we obtained solid descriptions of trust and distrust shopping experiences covering a variety of retail formats, such as drugstores, clothing stores, cosmetics stores, grocery stores, stationery stores, and supermarkets.

### Data Analysis Procedure and Results

#### Coding Process

In the first phase, we developed a coding frame to categorize critical incidents of trust. Drawing from previous studies on consumers’ trust in retail stores, we identified 37 antecedents under four dimensions (see Table 1).

**TABLE 1:** Coding frame and CIT results on antecedents of trust.

Antecedents	Source	Frequency of trust incidents	Frequency of distrust incidents
**1. Core product-related**		**55**	**65**
1.1 Product performance	Johnson and Grayson, 2005	39	53
1.2 Price/value	Orth and Green, 2009	12	0
1.3 Assortment	Guenzi et al., 2009	2	1
1.4 Ethical concern of manufacturers	Kennedy et al., 2001	0	0
*1.5 No discount price*		*2*	*0*
*1.6 Unreasonable label prices*		*0*	*11*
**2. Service-related**		**127**	**34**
2.1 After-sale service	Kim et al., 2005	42	18
2.2 Salespeople’s likability	Doney and Cannon, 1997	42	11
2.3 Salespeople’s competence	Sun and Lin, 2010	11	3
2.4 Salespeople’s benevolence	Kim et al., 2011	6	0
2.5 Salespeople’s similarity	Auh, 2005	5	0
2.6 Service complaint handling	Tax et al., 1998	4	2
2.7 Information quality	Fung and Lee, 1999;	3	0
2.8 Customization	Koufaris and Hampton-Sosa, 2004	2	0
2.9 Salespeople’s integrity	Kim et al., 2011	2	0
2.10 Convenience	Orth and Green, 2009	1	0
2.11 Open communication	Guenzi et al., 2009	1	0
2.12 Salespeople’s experience	Guenzi and Georges, 2010	1	0
2.13 Security control	Kim et al., 2008	0	0
2.14 Privacy policy	Kim et al., 2008	0	0
2.15 Customer orientation	Bejou et al., 1996	0	0
*2.16 Extra service*		*7*	*0*
**3. Third-party-related**		**1**	**10**
3.1 Third-party recognition	Siau and Shen, 2003	1	1
3.2 Institutional environmental trust	McKnight et al., 1998	0	0
3.3 External auditing	Siau and Shen, 2003	0	0
*3.4 Negative media coverage*		*0*	*9*
**4. Overall store-related**		**84**	**83**
4.1 Atmosphere	Lunardo and Mbengue, 2013	13	4
4.2 Integrity	Siau and Shen, 2003	13	69
4.3 Word of mouth	Ranaweera and Prabhu, 2003	10	0
4.4 Benevolence	Miyamoto and Rexha, 2004	9	3
4.5 Relationship quality	Miyamoto and Rexha, 2004	5	0
4.6 Advertisement	Aiken and Boush, 2006	4	0
4.7 Commitment	Miyamoto and Rexha, 2004	3	0
4.8 Country of origin	Kabadayi and Lerman, 2011	3	1
4.9 Reputation	Keh and Xie, 2009	3	0
4.10 Attractive rewards	Berry, 1995	3	0
4.11 Selling tactics	Kennedy et al., 2001	2	0
4.12 Competence	Siau and Shen, 2003	1	2
4.13 Familiarity	Kim et al., 2008	1	0
4.14 Responsibility	Pivato et al., 2008	0	0
4.15 Firm similarity	Johnson and Grayson, 2005	0	0
4.16 Community building	Siau and Shen, 2003	0	0
*4.17 National pride*		*4*	*0*
*4.18 Endorser*		*4*	*1*
*4.19 Firm spirits*		*2*	*1*
*4.20 Firm leaders*		*2*	*0*
*4.21 Operational format*		*1*	*0*
*4.22 Localness of retailers*		*1*	*0*
*4.23 Distrust in consumers*		*0*	*1*

*Italics indicate newly found antecedents not empirically examined in previous studies.*

In the second phase, two of the researchers independently classified each critical incident into antecedents of trust. If one incident that caused trust was not associated with any antecedent in the existing coding frame, a new antecedent was created and assigned under a corresponding dimension. The two researchers first randomly chose 20 incidents and independently read and coded them. Next, they met with an independent judge to compare the categorizations and address the discrepancies. Through this process, categorization methods, understanding of incidents, and interpretation of the antecedents in the coding frame converged. Next, the two researchers independently read and coded all incidents that led to trust (interrater reliability = 92%). The judge then met with them to compare the classifications; all disagreements were resolved through discussion. Finally, the same process was followed to code antecedents of distrust based on the coding frame developed in the first phase.

#### Antecedents of Trust

The CIT results (see Table 1) confirmed the majority of antecedents of trust that have been empirically validated in previous studies. Because these antecedents of trust have been tested in previous studies, we do not provide incidents to explain them. However, nine factors shown as antecedents of trust in previous studies (i.e., ethical concern of manufacturer, security control, privacy policy, institutional environmental trust, responsibility, similarity between firms and consumers, community building, external auditing, and salespeople’s customer orientation) did not emerge in our incidents of trust.

Finally, we uncovered eight new antecedents that have not been tested in previous studies: no discount price, extra services, national pride, endorsers, firm spirit, firm leaders, operational format, and localness of retailers. Extra services refer to on-site services that a retailer provides as a quick and improvised response to sudden changes in the environment. Firm spirit means that a firm has an inspiring and positive spirit. Table 2 lists the incidents of each newly found antecedent.

**TABLE 2:** New antecedents of trust and sample incidents.

Antecedents	Sample incidents
1.5 No discount price	“I’ve been feeling that specialty stores do not provide discount prices. ‘No discount price’ means ‘value for money’ for me. I feel embarrassed when haggling over price with salespeople. Even if the store discounts the price, I still feel lost. All in all, I trust the specialty stores.”
2.16 Extra service	“Finally, the extra and thoughtful service made me trust the store. The supermarket noticed the inconvenience of the consumers with children, so it provided childcare service for those consumers. I felt comfortable because the store put consumers’ benefits [first] when guaranteeing the safety of children.”
4.17 National pride	“I trust Lenovo because it is an exemplar of Chinese brands especially when it acquired the PC business of IBM. What a Chinese company, Lenovo did gain consumers’ trust because it made Chinese consumers feel extremely proud and excited.”
4.18 Endorsers	“I trust Watsons because its endorser is a basketball player in the [National Basketball Association]. I buy all my necessities in Watsons.”
4.19 Firm spirit	“After Liuxiang’s Olympic withdrawal in 2008, Nike launched an advertisement in which the attractive slogan and pictures represented the spirit–never give up. Through this advertisement, I could feel the spirit of Nike and thus trust Nike.”
4.20 Firm leaders	“I have trusted Lenovo since I watched an interview in which Chuanzhi Liu, the leader of Lenovo, introduced the processes of establishment, development, and overcoming [the] crisis of Lenovo and explained to the audience why he returned when the company faced difficulties. The whole story inspired me.”
4.21 Operational format	“In my opinion, such fast food chains as KFC must have a consistent pricing policy across franchises so I won’t be overcharged. Moreover, the sanitary conditions and the quality of food must be awesome. So, I trust KFC.”
4.22 Localness of retailers	“Sanjiang supermarket is a local company. Local supermarkets won’t do something harmful to local consumers. Because once they do so, it will be difficult for them to survive. Therefore, I trust Sanjiang supermarket among all the supermarkets.”

#### Antecedents of Distrust

The CIT results reveal 18 antecedents of distrust (see Table 3), three of which have not been examined in previous research: unreasonable label prices, negative media coverage, and firms’ distrust in consumers. Because antecedents of distrust have been underexplored in research, Table 3 provides incidents of all antecedents of distrust. The antecedent unreasonable label prices mean that consumers find a much higher price on the price tag than similar product prices of competitors. Because haggling over price is common in some Chinese markets, a high label price may signal that the retail store is trying to take advantage of consumers’ lack of information and is demonstrating malevolence. Therefore, consumers are likely to distrust stores with unreasonably high label prices. Negative media coverage means that the media discloses negative information about firms to consumers, which may lead consumers to distrust the firms. Finally, firms’ distrust in consumers means that firms suspect that consumers will steal products from the store. For example, some self-service stores frisk consumers before allowing them to enter, thus signaling distrust in consumers. In turn, consumers may distrust the stores.

**TABLE 3:** Antecedents of distrust and sample incidents.

Antecedents	Sample incident
**1. Core product-related**	
1.1 Product performance	“When I was a freshman, I liked dumplings in a restaurant nearby campus. Once, I went there to eat dumplings with pork stuffing. After eating the dumplings, I felt indisposed and had a stomachache. My mom said I was slightly poisoned. I felt very shocked and decided not to go to that restaurant forever. Since then I have distrusted that restaurant.”
1.3 Assortment	“Last year I entered a store named Metersbonwe with my friends. The clothes were very outdated perhaps because of the looks of the clothes on the shelves. Suddenly, I felt disappointed. Since then, I have distrusted Metersbonwe.”
*1.6 Unreasonable label prices*	“Finally, we found that product [we’ve been wanting] in the store. However, the label price was shockingly low—only ¥10. I was really shocked and wondered whether it was a counterfeit. Then I searched for information online and confirmed that the average price of this product was way higher than ¥10. Although I was not sure whether it was a counterfeit, I have distrusted that store since then.”
**2. Service-related**	
2.1 Poor after-sale service	“I once bought a pair of flip-flops at a store named ‘Specialty.’ What astounded me was that the flip-flops were broken after being worn 10–20 minutes. I went back to the store to return the shoes. But the salesperson denied the return and insisted that she never sold those shoes. I have distrusted that store since then.”
2.2 Unlikable salespeople	“I wanted to try on the clothes before making a purchase decision. But the salesperson told me that I was not allowed to try them on if I did not plan to buy. I said nothing and decided never to go to that store. I distrusted the store because the salesperson was arrogant.”
2.3 Salesperson’s incompetence	“Last semester, I went to a cosmetics store to buy facial cleaner. The salesperson was very thoughtful and recommended to me a product according to my skin condition. But my face started to swell up after using it for two weeks. I returned the product and got my money back. However, I have lost my trust in the store because I think the salesperson was … incompetent–she did not recommend to me the right product for my skin.”
2.6 Ignore service complaints	“I went to a barbershop to trim my hair. After the trimming, I found that the hairstyle was not what I wanted and required the hairstylist to redo it. But he ignored my request, making me angry. Since then I have never patronized that barbershop and have distrusted the hairstylist and the store.”
**3. Third-party-related**	
3.1 Negative third-party evaluation	“I had always had dinner in a restaurant in the campus cafeteria. One day, I met a friend who told me that our university checked the sanitation of all restaurants in the cafeteria and found that the restaurant I usually go to has the worst sanitation. Since then, I have distrusted the restaurant and never been there for dinner.”
*3.4 Negative media coverage*	“Recently, some media has reported that KFC added additives and preventatives into food, sold food past sell-by dates, and purchased low-quality materials, making me distrust KFC.”
**4. Overall store-related**	
4.1 Poor atmosphere	“Once I went to a Café to have a rest. I felt the atmosphere was awesome and relaxing when I just entered. But I suddenly saw a mouse when sitting there. At that time, I thought it was not a big deal. When I was leaving, I saw their kitchen. What a mess! Various unwashed utensils stood there, and the trash was full of garbage. Therefore, I thought: It’s not because the mouse wants to come, but they had created an appropriate environment for the mouse. So, I have never been there since because I do not believe the grandeur on the surface.”
4.2 Improbity	“I guess the reason that I lost my trust in Carrefour was because of the policy of the refund of the five-times price difference. What occurred to me was that I was charged ¥8.9 for the product with a label price of ¥6.9. The shop assistant, however, just gave two options: either return the product or return the ¥2 and did not mention the refund policy of the five-times price difference. I know that Carrefour has tried its best to make some changes, but I have lost my trust in it.”
4.4 Malevolence	“I thought it was a good deal to buy two yogurts and get two for free. However, after arriving at home, I found the expired date was just today! I was very angry. Under normal circumstances, they only give you one yogurt for free, but two were given for free just because the yogurt was about to expire. I think Walmart sells yogurt that’s about to expire and ignores customers’ feelings, which made me no longer trust Walmart.”
4.8 National brand was sold abroad	“Last semester, a professor told me that the brand of China a toothpaste brand was sold overseas. At that time, I felt deceived and fooled. From then on, I have never bought the toothpaste of the brand of China.”
4.12 Incompetence	“After searching for information, I recognized that Lining [a sports brand] operated ineffectively, reformed unsuccessfully, raised products’ prices due to failures in cost control, and had an irrational human resource structure. Therefore, I had a bad image of Lining. Since then, I have distrusted Lining and never again bought its products.”
4.18 Dislike endorser	“Since [a celebrity] became the endorser of OLAY, I have distrusted OLAY because I hated this person, and I thought that her skin was not that good.”
4.19 Lack of firm spirit	“After Reebok and Adidas merged, I found the personality and characteristics of Reebok had vanished. The previous spirit ‘I am what I am’ disappeared without a trace. I was very disappointed. Since then, I have always distrusted Reebok and have never patronized any Reebok store.”
*4.23 Distrust in consumers*	“I was very disappointed after shopping there. First of all, customers were not allowed to carry in their bags, a signal of distrust in customers, in my opinion. In addition, it brings unnecessary troubles to customers.”

*Italics represent antecedents not empirically examined in previous studies.*

According to the results, the top three antecedents of distrust—improbity (35.9%), unacceptable product performance (27.6%), and poor after-sale service (9.38%)—account for a large proportion of reasons to distrust. Improbity is when a company acts on rules that consumers do not accept, such as promise breaches, cheating, adulteration, jerry-building, and deception. Unacceptable product performance includes two dimensions: unacceptable product quality and unsatisfactory performance in use. The former means that the quality is below the industry standard, while the latter means that the product reaches the industry standard but is below consumers’ expectations. Poor after-sale service means that stores do not provide satisfactory after-sale services, such as free returns.

#### Comparison of Antecedents of Trust and Distrust

The CIT results show that only 14 of 37 antecedents of trust constitute the antecedents of distrust as well. In other words, if a store is doing well in these 14 elements, consumers’ trust in the store will increase. Otherwise, consumers’ distrust will increase. Moreover, we find that three antecedents of distrust may not influence Chinese consumers’ trust: label prices, media coverage, and firms’ trust/distrust in consumers. In other words, consumers may not trust a store just because of a reasonable price tag, positive media coverage, or the firm’s trust in consumers. However, these factors can lead to consumer distrust. In summary, the results suggest that the antecedents of trust and distrust overlap only minimally.

Drawing on Oliver’s (2014) framework, we classify the antecedents into three categories (see Table 4): essential attributes, bivalent attributes, and psychological-extra attributes. Essential attributes are “fundamental but unprocessed attributes capable of causing ill feelings when flawed” (Oliver, 2014, p. 149). In our study, we find three essential attributes: unreasonable label prices, negative media coverage, and distrust in consumers. Bivalent attributes are “those upward and downward translatable attributes that can cause” both ill feelings and good feelings (Oliver, 2014, p. 149). We find 14 bivalent attributes, such as product performance, which increases trust (vs. distrust) if firms are doing well (vs. badly) on these attributes. Finally, psychological-extra attributes are means to fulfill consumers’ needs beyond a functional level but engender good feelings only when superb (Oliver, 2014). However, when these psychological-extra attributes are not superb, consumers will not necessarily have bad feelings about the firms. Our results reveal 24 psychological-extra antecedents, such as customization, convenience, outstanding leaders of a firm, and so on. In summary, among the factors affecting trust and distrust, most are psychological-extra attributes. Therefore, we can conclude that many antecedents leading to trust may not affect distrust if a retail store is doing a poor job with the antecedents, implying that building trust does not mean the elimination of distrust.

**TABLE 4:** Categories of antecedents of trust and distrust.

Categories	Antecedents
Essential attributes (only mentioned	1.6 Unreasonable label prices
in distrust incidents)	3.4 Negative media coverage
	4.23 Distrust in consumers
Bivalent attributes (mentioned in both	1.1 Product performance
trust and distrust incidents)	1.3 Assortment
	2.1 After-sale service
	2.2 Salespeople’s likability
	2.3 Salespeople’s competence
	2.6 Service complaint handling
	3.1 Third-party recognition
	4.1 Atmosphere
	4.2 Integrity
	4.4 Benevolence
	4.8 Country of origin
	4.12 Competence
	4.18 Endorser
	4.19 Firm spirits
Psychological-extra attributes	1.2 Price/value
(only mentioned in trust incidents)	1.5 No discount price
	2.4 Salespeople’s benevolence
	2.5 Salespeople’s similarity
	2.7 Information quality
	2.8 Customization
	2.9 Salespeople’s integrity
	2.10 Convenience
	2.11 Open communication
	2.12 Salespeople’s experience
	2.16 Extra service
	4.3 Word of mouth
	4.5 Relationship quality
	4.6 Advertisement
	4.7 Commitment
	4.9 Reputation
	4.10 Attractive rewards
	4.11 Selling tactics
	4.13 Familiarity
	4.17 National pride
	4.20 Firm leaders
	4.21 Operational format
	4.22 Localness of retailers

## Discussion

This study relies on CIT to identify and compare the antecedents of Chinese consumers’ trust and distrust. The results show that trust and distrust share antecedents to a minimum degree. Therefore, this study makes several contributions to the literature and offers insightful implications for marketers.

First, through CIT, we find eight underexamined antecedents of Chinese consumers’ trust: no discount price, extra services, national pride, endorsers, firm spirit, firm leaders, operational format, and localness of a retailer. Consistent with research on emotional trust (e.g., McAllister, 1995), many of these factors, such as endorsers, national pride, and firm spirit, are related to emotions; however, they may not directly lead to consumer trust. For example, one of the reasons consumers trust a chain store (i.e., operational format) is that they believe that the store is resourceful and thus that the retailer has no need to engage in unethical activities to gain profits. That is, the operational format may influence consumers’ trust through their evaluation of firms’ integrity. In addition, consumers often trust local firms because they believe such firms would have to pay a higher cost in the local market than national firms if unethical activities were found. Further research is necessary to test the mechanism through which these factors influence consumer trust.

Second, the results show three antecedents of distrust that have not been empirically examined in previous studies: unreasonable label price, negative media coverage, and distrust in consumers. Price usually functions as a cue when consumers are evaluating product quality, store image, and store trustworthiness, especially when they do not have sufficient internal cues of a store. However, extant studies have overlooked the effect of unreasonable label prices on stores’ image or distrust. As noted previously, when the label price of a product is unreasonably higher than similar products in the market, consumers will likely judge the store as trying to take advantage of consumers who are poor at negotiations. In this case, consumers will question the store’s benevolence and integrity, leading to distrust.

Third, this research adds more qualitative evidence to the notion that trust and distrust are two distinct constructs rather than bipolar constructs. Before Lewicki et al. (1998) proposed trust and distrust as distinct constructs, scholars generally held a unidimensional view that trust and distrust are bipolar and treated high distrust as indicative of the absence of trust (e.g., Rotter, 1971; Johnson-George and Swap, 1982; Tardy, 1988; Govier, 1994). Influenced by the unidimensional view, some contemporary studies have continued to use trust/distrust as a bipolar item in measurement (e.g., Wang and Lee, 2018). As Lewicki et al. (1998) indicated, identifying the relationship between trust and distrust should be helpful if studies can show that trust and distrust do not share all the same antecedents. According to this logic, the CIT results reveal that the antecedents of trust outnumber those of distrust, and some factors only appeared in either trust or distrust incidents of participants. These findings imply that some marketing activities that lead to trust may not necessarily eliminate consumers’ distrust. Thus, treating trust and distrust as two ends of a continuum might be too simplistic.

Finally, the results provide retailers with guidance on managing customer relationships. Because our findings indicate that consumers do not necessarily trust a retailer even when they do not distrust it, retailers still need to pay attention to both improving consumers’ trust and eliminating distrust. On the one hand, retailers should leverage cues that generate trust to maximize consumers’ trust. On the other hand, retailers should endeavor to eradicate cues that lead to distrust, so that consumers will trust a retailer without uncertainty and suspicion and thereby be more likely to purchase from the store. These findings are especially insightful, as, with online shopping booming, increasingly less research attention has been paid to physical retail stores. As the industry with the most employees, retailing deserves more research attention in this area. Future research could also compare trust and distrust in other contexts in which trust is critical, such as financial services, business-to-business relationships, and medical care services.

### Limitations and Future Research

Our study suffers from a few limitations that provide avenues for future research. First, we did not ask participants to focus on one specific retail format. Covering a variety of retail formats helps provide a more comprehensive set of antecedents, but it also may have led participants to confuse store trust with brand trust. For example, some participants articulated their trust in the Nike brand instead of a specific Nike retail store. However, consumers likely do not indistinguishably trust all Nike retail stores. As for franchisees of a retail store brand trust and store trust are difficult to differentiate, future research should investigate what factors influence brand trust and what factors influence store trust.

Second, we discovered antecedents of trust and distrust by reviewing previous research and CIT, but the relationships between these antecedents need more scrutiny using experiments. For example, consumers may believe that stores providing value-added services are trustworthy because they think such stores are responsible. Future research could use experiments to examine the relationship between these antecedents and their interactive effects on trust and distrust to better understand trust and distrust.

Third, order effects may have reduced the validity of the results. During the data collection process, we asked the participants to write a story about a trust experience followed by a distrust experience. Doing so might have biased participants’ memories such that they might have tried to write a distrust story completely distinct from their trust story. For example, if they wrote a trust story about product quality, they might have believed that the researchers expected some other factors that influence distrust, such as salespeople. As a result, the retrieval of memory about a distrust experience might have been biased by the trust story.

Finally, some antecedents of trust that are well documented in extant literature based in Western countries did not emerge in the current study. Therefore, future research might investigate what factors lead Chinese consumers to have different formation mechanisms than those of consumers from other countries.

## Data Availability Statement

The original contributions presented in the study are included in the article/supplementary material, further inquiries can be directed to the corresponding author/s.

## Ethics Statement

The studies involving human participants were reviewed and approved by the Academic Committee of Center for Business School of Jilin University. Written informed consent for participation was not required for this study in accordance with the national legislation and the institutional requirements.

## Author Contributions

All authors listed have made a substantial, direct and intellectual contribution to the work, and approved it for publication.

## Conflict of Interest

The authors declare that the research was conducted in the absence of any commercial or financial relationships that could be construed as a potential conflict of interest.
